# Toxic Substances Control Act (TSCA) Implementation: How the Amended Law Has Failed to Protect Vulnerable Populations from Toxic Chemicals in the United States

**DOI:** 10.1021/acs.est.2c02079

**Published:** 2022-08-18

**Authors:** Swati D.G. Rayasam, Patricia D. Koman, Daniel A. Axelrad, Tracey J. Woodruff, Nicholas Chartres

**Affiliations:** † Program on Reproductive Health and the Environment, Department of Obstetrics, Gynecology and Reproductive Sciences, University of California San Francisco School of Medicine, San Francisco, California 94143, United States; ‡ Environmental Health Sciences, University of Michigan School of Public Health, Ann Arbor, Michigan 48109, United States; § Independent Consultant, Washington, D.C. 20004, United States; ∥ Environmental Research and Translation for Health, Department of Obstetrics, Gynecology and Reproductive Sciences, University of California San Francisco School of Medicine, San Francisco, California 94143, United States

**Keywords:** environmental health, risk assessment, hazard identification, federal policy, susceptibility, environmental justice, health equity

## Abstract

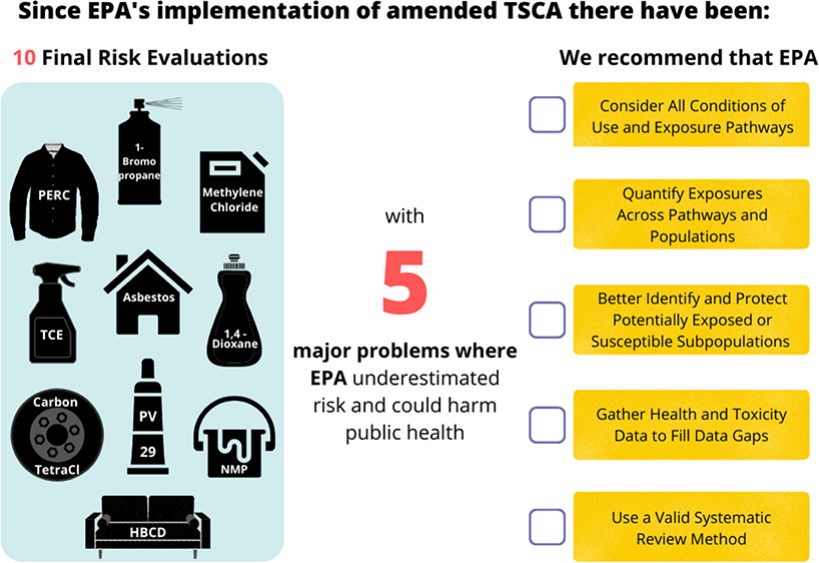

Exposures to industrial chemicals are widespread and can increase the risk of adverse health effects such as cancer, developmental disorders, respiratory effects, diabetes, and reproductive problems. The amended Toxic Substances Control Act (amended TSCA) requires the U.S. Environmental Protection Agency (EPA) to evaluate risks of chemicals in commerce, account for risk to potentially exposed and susceptible populations, and mitigate risks for chemicals determined to pose an unreasonable risk to human health and the environment. This analysis compares EPA’s first 10 chemical risk evaluations under amended TSCA to best scientific practices for conducting risk assessments. We find EPA’s risk evaluations underestimated human health risks of chemical exposures by excluding conditions of use and exposure pathways; not considering aggregate exposure and cumulative risk; not identifying all potentially exposed or susceptible subpopulations, and not quantifying differences in risk for susceptible groups; not addressing data gaps; and using flawed systematic review approaches to identify and evaluate the relevant evidence. We present specific recommendations for improving the implementation of amended TSCA using the best available science to ensure equitable, socially just safeguards to public health. Failing to remedy these shortcomings will result in continued systematic underestimation of risk for all chemicals evaluated under amended TSCA.

## Abstract 2

EPA’s implementation of amended TSCA did not incorporate key scientific/methodological factors, underestimating health risks of chemical exposures, particularly to susceptible populations.

## Introduction

The 1976 Toxic Substances Control Act (TSCA) was enacted in response to the growing incidence of “environmental disease” caused by the boom in industrial chemical manufacture after World War II.^1^ Then U.S. Environmental Protection Agency (EPA) Administrator Russell Train called TSCA “one of the most important pieces of “preventive medicine” legislation” ever passed by Congress.^2^ In 1979, the President’s Toxic Substances Strategy Committee concluded chemicals were a significant source of death and disease in the U.S. and “measured against the need, the handful of chemicals regulated to date have been disappointingly small”.^3^ TSCA remains the primary authority in the U.S. regulating nonpesticide chemicals in commerce.^4^

Since 1976, global concerns regarding chemical risks have grown. Recent estimates by the World Health Organization identify two million lives and fifty-three million disability-adjusted-life-years were lost worldwide in 2019 due to chemical exposures.^5^ There is now significantly more evidence on chemical exposures and risks, and increased attention to disproportionate risks to populations near polluting facilities (fenceline communities), children, consumers, and workers.^6,7^

Under 1976 TSCA, chemicals already in commerce were assumed to be safe until shown harmful, and the original law was widely viewed as weak and ineffective.^4,8,9^ TSCA provided limited authority to obtain necessary information to assess the risks of chemicals, and imposed significant barriers to regulating chemicals posing substantial risks, even for substances with known harms, such as asbestos.^8−13^ Various state and local jurisdictions enacted their own chemical laws and regulations to partially fill the gaps left by TSCA.^14,15^ Increasing market globalization, accumulating scientific evidence of risk, and the growing patchwork of federal, state, and local regulatory requirements eventually set the stage to update TSCA via the Frank Lautenberg Chemical Safety for the 21st Century Act (amended TSCA), enacted in June 2016.^16^ In the 40 years between enactment of original TSCA and its 2016 amendments, EPA regulated fewer than 10 of over 86 000 existing chemicals registered for use in commerce.^17,18^

Amended TSCA requires EPA to conduct risk evaluations of chemicals in commerce on a specified schedule, consider risks to “potentially exposed or susceptible subpopulations” (PESS), and determine if a chemical poses an “unreasonable risk” without consideration of cost.^19^ It also requires EPA to regulate any existing chemical determined to pose an unreasonable risk “to the extent necessary so that the chemical substance or mixture no longer presents such risk”.^19^ Finally, it requires EPA to “use scientific information, technical procedures, measures, methods, protocols, methodologies, or models, employed in a manner consistent with the best available science”.^19^

However, the amended law is missing aspects of its regulatory contemporaries in the generation and use of scientific data. Unlike the European Union’s Registration, Evaluation, Authorisation and Restriction of Chemicals (REACH), amended TSCA still places the burden of obtaining the necessary data to evaluate existing chemicals on EPA rather than manufacturers.^20^ Amended TSCA also lacks important scientific principles found in the Food Quality Protection Act (FQPA) of 1996, including requirements for EPA to apply adjustment factors to account for early life susceptibility and calculate aggregate exposure and cumulative risk.^21^ Further, amended TSCA preempts some state action and concentrates authority at the federal level barring some exceptions (such as California’s Proposition 65).^15^ (see Supporting Information (SI) Section 1) This leaves many critical implementation decisions with EPA, including how to assess and apply the available science, making it vulnerable to political interference and scientific integrity concerns.^22−24^ The weaknesses in amended TSCA could be improved by health-protective implementation of EPA’s existing authorities. The stakes are high, as widespread use of industrial chemicals, many of which can cross the placental barrier, has led to generations of children being born prepolluted.^16,25^

How EPA utilizes science to implement amended TSCA is important to population health, particularly to PESS. In this analysis, we compare EPA’s first 10 chemical risk evaluations completed under amended TSCA between June 2020 and January 2021 (referred to as the “first 10”) to the “best available science” to evaluate risks to public health from chemicals in commerce.^26^ The first 10 chemicals are asbestos, 1-bromopropane (1-BP), carbon tetrachloride, C.I. pigment violet 29 (PV29), hexabromocyclododecane (HBCD), 1,4-dioxane, methylene chloride, *N*-methylpyrrlidone (NMP), perchloroethylene (PCE), and trichloroethylene (TCE).

We first present an overview of key provisions in TSCA regarding prioritization and risk evaluation. We then review EPA’s approach to several elements common to all TSCA risk evaluations:

- Conditions of use and exposure pathways,
- Aggregate exposure and cumulative risk,
- Potentially exposed or susceptible subpopulations (PESS),
- Data gaps, and
- Systematic review.

We selected these topics based on our previous studies and their importance to estimation of risk.^11,27^ For each element we discuss (1) what is required under amended TSCA, (2) how EPA implemented these requirements during the first 10 risk evaluations, (3) the public health implications of EPA’s implementation, and (4) our recommendations if the element is scientifically inadequate. This paper does not cover all the issues with the first 10 risk evaluations, but other manuscripts discuss additional critical issues including using health-protective adjustment (uncertainty) factors for risk characterization and a unified approach to dose–response assessment.^28−30^

## Overview of the TSCA Risk Evaluation Process

Under amended TSCA, EPA must develop processes to evaluate and address risks to human health and the environment from “New Chemicals” (chemicals not yet on the market) and “Existing Chemicals” (chemicals currently on the market in the U.S.) (see SI Section 2 and Figure S1). This analysis focuses on EPA’s existing chemicals risk evaluations.

Amended TSCA requires EPA create a process designating existing chemicals as either “high-priority” (requiring risk evaluation) or “low-priority” substances (risk evaluations not currently required) (see SI Section 2 and Figure S2).

Amended TSCA requires EPA take two initial actions to evaluate the first set of existing chemicals. First, EPA had to select 10 existing chemicals for evaluation by December 2016 and complete those evaluations by June 2020. Second, EPA was required to issue final “framework” rules outlining its approach for chemical prioritization and risk evaluation by June 2017. The framework rules were proposed in January 2017 by the Obama-Biden EPA but finalized in July 2017 by the Trump-Pence EPA. Some deficits in EPA’s risk evaluations described below are a result of changes between proposed and final versions of the framework rules; relevant changes are outlined in SI Section 3.

## Conditions of Use and Exposure Pathways

Defining how chemicals are used and how people come into contact with them is a key to identifying exposures and risks.

## What Is Required under Amended TSCA

The law outlines several requirements for the contents of a risk evaluation (SI Section 1). EPA must:

integrate and assess available information on hazards and exposures for the conditions of use of the chemical substance §2605(b)(4)(F)(i);
take into account, where relevant, the likely duration, intensity, frequency; and number of exposures under the conditions of use of the chemical substance §2605(b)(4)(F)(iv)
“conditions of use” means the circumstances. . .under which a chemical substance is intended, known, or reasonably foreseen to be manufactured, processed, distributed in commerce, used, or disposed of §2602(4).

Taken together, these passages require EPA comprehensively assess conditions of use and exposures pathways in its risk evaluations; affirmed by a 2019 appeals court ruling.^31,32^ The only statutory exclusions are for certain uses regulated under other statutes, such as pesticides, tobacco products, food additives, and cosmetics.

## How EPA Implemented These Requirements

EPA’s first 10 risk evaluations addressed exposures from a broad range of conditions of use through multiple exposure pathways; however, the Agency excluded several aspects of exposure based primarily on two inappropriate rationales.

1. EPA asserted it could choose which conditions of use to include in each risk evaluation. In its final risk evaluation framework rule, EPA claimed it could exclude certain uses, asserting broad discretion to select conditions of use to assess “for each chemical substance on a case-by-case basis. . .consistent with the objective of conducting a technically sound, manageable evaluation.”^33^

This claim of discretion to exclude conditions of use substantially affected the scope of three out of the first 10 risk evaluations: asbestos, carbon tetrachloride, and 1,4-dioxane (see SI Section 2 and Table S1). For example, EPA’s final “Asbestos Part 1” considers only current uses, excluding ongoing exposures from legacy uses (e.g., past uses of asbestos, as in automotive brakes or housing materials, that can result in current exposure) and associated disposal.

2. EPA limited the exposures considered in risk evaluation by interpreting TSCA as only considering chemical exposures not addressed by other environmental statutes, rather than a comprehensive chemical risk reduction tool. In May 2018, EPA issued problem formulation documents for each TSCA risk evaluation saying EPA would "focus its analytical efforts on exposures that are likely to present the greatest concern and consequently merit a risk evaluation under TSCA, by excluding, on a case-by-case basis, certain exposure pathways that fall under the jurisdiction of other EPA-administered statutes.”^34^

EPA’s decision to narrowly limit exposure pathways considered under TSCA had a substantial impact on the first 10 risk evaluations. In eight, the Agency did not assess three or more exposure pathways such as ambient air, disposal, or drinking water, based on the rationale of being addressed by other statutes like the Clean Air Act (CAA), Safe Drinking Water Act (SDWA), or Clean Water Act (CWA) (SI Section 2 and Table S2).

## Implications for Public Health

EPA’s exclusions of conditions of use in three of the first 10 risk evaluations and exposure pathways in eight of the first 10 mean these evaluations systematically underestimated exposure and risk. The logic of assuming that coverage by another statute results in sufficient risk reductions is flawed as it requires EPA to assume equal levels of protection from different statutes. Although other statutes such as the CAA and SDWA may have some overlapping jurisdiction, they do not necessarily meet the health-protective standards required by amended TSCA. Under CAA, EPA evaluates residual risk for chemicals specified as hazardous air pollutants (HAPs) following implementation of technology-based standards. However, these residual risk analyses have gaps and limitations, for example EPA is not required to consider risks of combined emissions from different industries to fenceline communities. The reduction of some chemicals under other statutes can result in regrettable substitutions and less health-protective outcomes than TSCA.^35^ For example, EPA’s Regulatory Impact Analysis (RIA) for reductions of hydrofluorocarbons (HFCs) under the American Innovation and Manufacturing Act of 2020 (AIM Act) documented that increased production of hydrofluoroolefins (HFOs), expected to substitute for HFCs could increase carbon tetrachloride emissions. Deferring risk management of these emissions to other statutory authorities which, unlike TSCA, do not contain explicit language to consider risks to PESS could result in increased risks in communities already experiencing elevated respiratory and cancer risks.^36^

EPA’s exclusions also involved instances where a chemical was not regulated, even though it was within jurisdiction of another statute, which is inconsistent with EPA’s justification for exclusion. For example, EPA’s 1-bromopropane risk evaluation, finalized in August 2020, did not assess the ambient air pathway, even though 1-bromopropane was not listed as a HAP until January 2022, and any new or revised CAA standards for industry sectors emitting 1-bromopropane may not be established for several years.^37^ EPA estimates 1-bromopropane is in widespread use, with annual 2007 emissions of 20 000 to 30 000 t and with a growth rate of up to 20%/year in the U.S.^38^ EPA’s 1,4-dioxane risk evaluation similarly excluded the drinking water pathway, even though under the SWDA, EPA has not established a National Primary Drinking Water Regulation for 1,4-dioxane or even decided whether one is necessary. Almost 30 million people in the U.S. receive drinking water with 1,4-dioxane levels above the reference concentration of 0.35 μg/L.^39^

TSCA, unlike other statutes, offers the opportunity for primary prevention (eliminating risk at the source), which can be more effective than regulatory tools available under other statutes and has been promoted as an EPA strategy since the 1990s.^40,41^ For example, it may be more effective and less costly to use TSCA to prevent releases of certain chemicals (such as 1,4-dioxane) to water, rather than trying to use the SDWA and CWA to address water contamination after the fact. EPA can only determine whether regulations under other statutes are sufficient to meet TSCA’s “unreasonable risk” determination by assessing all conditions of use and exposure pathways in the risk evaluation first. In addition, even when exposures are within jurisdiction of other statutes they may be important contributors to aggregate exposures (see discussion below in Aggregate Exposure and Cumulative Risk) and affect the determination of whether or not a chemical poses an unreasonable risk

EPA’s exclusion of conditions of use and exposure pathways from risk evaluations may pose disproportionate risks to PESS. For example, communities near manufacturing facilities and contaminated sites are often those with lower wealth, poorer health, and with a majority of residents who are people of color.^42−44^ Chemical exposures from industry emissions to air and releases to water frequently result in disproportionate exposures to these communities, even after accounting for regulatory controls under other statutes, particularly as communities of color are more likely to have water systems with repeat violations under the SDWA, leading to higher exposures.^45−47^ As TSCA has an explicit charge to consider PESS (discussed below), it is important to consider how conditions of use and exposure pathways pose risks to overburdened communities, as it allows EPA to make informed decisions about how to best regulate.

## Recommendations for Change

EPA should revise its first 10 risk evaluations to incorporate all conditions of use and include exposure pathways within the jurisdiction of other EPA statutes and continue to do so in future risk evaluations.

In June 2021, EPA announced it would conduct further analysis of at least some of the excluded exposure pathways for seven of the first 10 chemicals, and it would also revisit the excluded 1,4-dioxane byproduct conditions of use.^133^ In January 2022, EPA released a draft “screening level methodology” for assessing the air and water pathways.^44^ Following completion of this methodology, EPA will consider whether to revise or supplement the risk evaluations to account for currently excluded exposure pathways.

## Aggregate Exposure and Cumulative Risk

Failure to assess aggregate exposure and cumulative risk results in evaluations that understate exposure and risk. EPA has assessed aggregate exposure and cumulative risk of pesticides as required by the 1996 Food Quality Protection Act, but it has rarely done such analyses of industrial chemicals under TSCA.^21^

## What Is Required under Amended TSCA

When conducting a risk evaluation, amended TSCA requires the EPA (SI Section 1) to:

describe whether aggregate or sentinel exposures chemical substance under the conditions of use were considered, and the basis for that consideration §2605 (b)(4)(F)(ii).

Amended TSCA also requires EPA to eliminate the unreasonable risk posed by a chemical substance from:

the manufacture, processing, distribution in commerce, use, or disposal of a chemical substance or mixture, or any combination of such activities §2605 (d)(3)(A)(i)(I).

EPA defines aggregate exposure as “the combined exposures to an individual from a single chemical substance across multiple routes and across multiple pathways,”^33^ and cumulative risk assessment as the “analysis, characterization, and possible quantification of the combined risks to health or the environment from multiple agents or stressors,” considering both chemical and nonchemical stressors.^49^ Nonchemical stressors include biological and physical agents (e.g., pathogens), psychosocial stressors (e.g., exposure to fatal police violence), and health or disease status (e.g., diabetes).

## How EPA Implemented These Requirements

**Figure 1:**
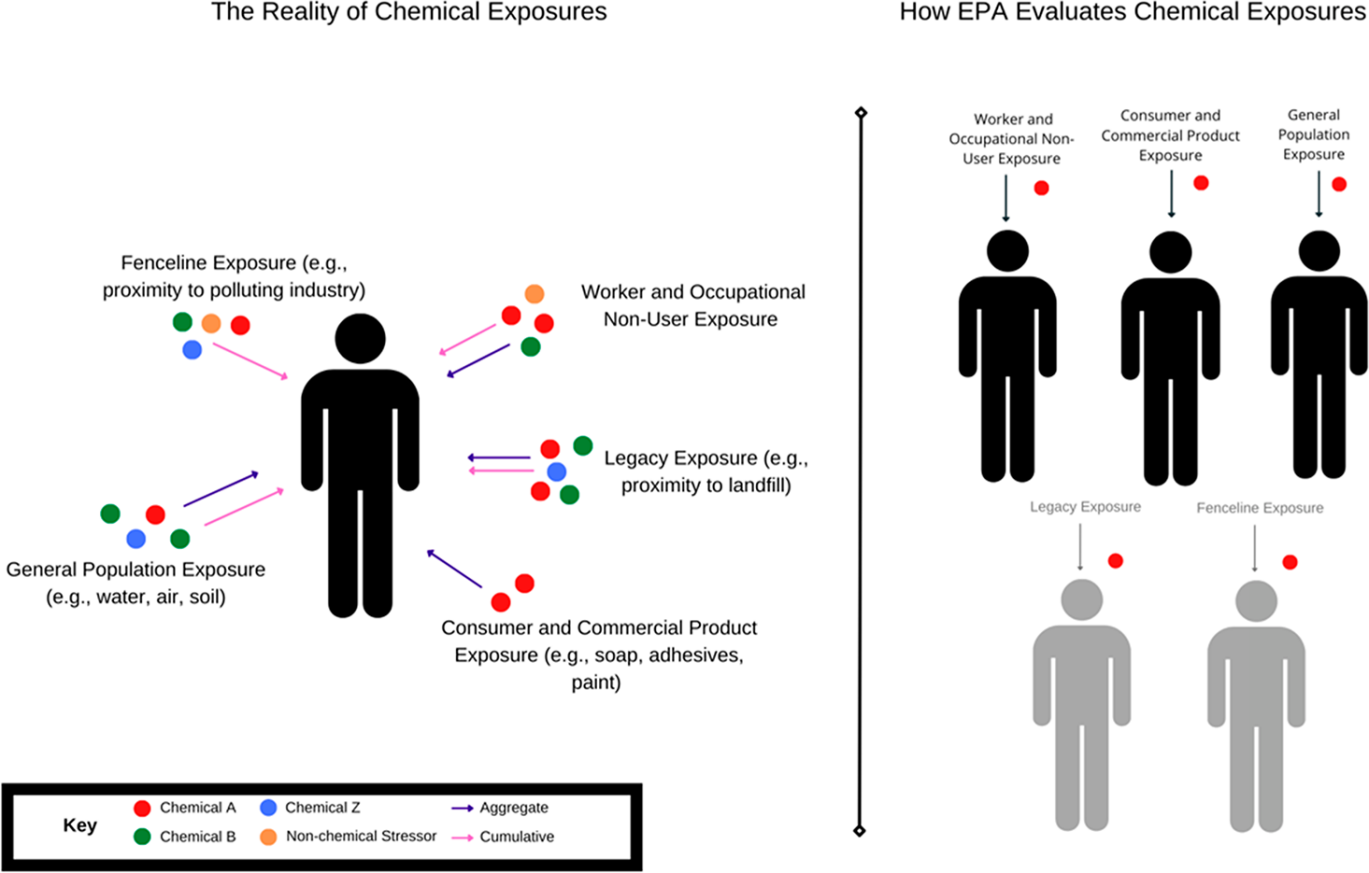
An example of aggregate and cumulative exposure to chemicals and nonchemical stressors across sources and populations compared to the current EPA approach. Though not shown, within these exposure pathways, EPA separated individual consumer or commercial product uses by product type and separated workers and what EPA refers to as occupational nonusers (those in the workplace exposed but not using the chemical under evaluation). The figures in gray represent the pathways that EPA has yet to implement under amended TSCA.

EPA, to a limited extent, considered aggregate exposure in two of the first 10 risk evaluations. For NMP, EPA aggregated dermal and inhalation exposure using a pharmacokinetic model but did not consider combined uses or exposure settings (e.g., both at work and at home).^50^ For HBCD, EPA aggregated general population exposures to environmental media using population biomonitoring data.^51^ In the remaining eight risk evaluations, EPA considered sentinel rather than aggregate exposures due to concerns about “overestimating” risk, as detailed below.^52^ EPA assessed three exposed populations separately: workers exposed directly or indirectly; consumers exposed via products; and the general population exposed via ambient air and drinking water. However, EPA assessed inhalation and dermal exposures separately for workers, without calculating combined exposure for workers exposed via both routes. EPA also assessed consumer exposures for individual products without calculating the combined exposure for consumers using multiple products containing the same chemical. Finally, EPA did not aggregate the exposures of individuals who have occupational, consumer, and general population exposures, such as individuals exposed at both work and home (Figure 1).

EPA did not conduct cumulative risk assessments in the first 10 risk evaluations, preventing consideration of how chemical exposure risks may be amplified by coexposure to other chemicals contributing to common adverse outcomes or to nonchemical stressors, such as antiblackness or xenophobia, exacerbating the risk of adverse outcomes from chemical exposures.^53,54^

## Implications for Public Health

In the U.S., more than 130 million people reside in “vulnerability zones” or communities surrounding one or more of the 3433 facilities producing, storing, and using highly toxic chemicals, the majority of these are Black or Latino and have higher than average rates of poverty.^55^ These communities have not been adequately protected from environmental harms, with even the most fundamental protections afforded under the law.^56−58^

EPA’s choice to consider sentinel and not aggregate exposures underestimated risk in the first 10 risk evaluations, as we illustrate using 1,4-dioxane. A worker may inhale and be dermally exposed to a 1,4-dioxane solvent during their shift and exposed at home through multiple consumer products, such as shampoos and all-purpose cleaners containing 1,4-dioxane. However, EPA calculated worker risks separately for inhalation and dermal exposures and separately for each consumer product without considering the exposure of workers who are also consumers. This worker may also live near their workplace, a factory releasing 1,4-dioxane into the air and drinking water, but EPA’s risk evaluation did not consider drinking water or ambient air exposures (see Conditions of Use and Exposure Pathways above, and SI Section 2 and Table S2). A complete aggregate exposure assessment would account for individuals who experience combinations of inhalation and dermal exposure at work, contact with multiple consumer products at work or home, and are exposed to contaminated air or drinking water in their communities. EPA indicated that “Using an additive approach to aggregate exposure and risk in this case would result in an overestimate of risk” without providing evidence to support this assertion.^52^ EPA’s concern about *over*estimation of risk led to the Agency addressing exposures independently, resulting in *under*estimation of risk to individuals exposed via multiple pathways, multiple settings, and multiple conditions of use (Figure 1); failing to meet its mandate to protect public health and in particular PESS who disproportionately experience these overlapping exposures.

Not considering cumulative risk also underestimates risk.^59,60^ For example, using publicly available data from the U.S. EPA Toxics Release Inventory (TRI), researchers were able to establish counties throughout the U.S. reporting air emissions of various chemicals linked to respiratory cancer, including formaldehyde, a leukemogen. Nineteen counties were identified with a total of 10 or more respiratory carcinogens being reported (including formaldehyde) and an analysis of the demographic characteristics of these counties found correlations between the number of facilities releasing formaldehyde air emissions and speaking English “less than well”, living in a single-parent household, living in a mobile home, living in multiunit housing, or identifying as having a disability.^61^ By only examining the risk of an adverse outcome from exposure to a single chemical, EPA overlooked how multiple exposures (chemical and nonchemical) may combine to produce a common adverse health outcome. (Figure 1).

## Recommendations for Change

Only amended TSCA provides the ability to aggregate exposure of a single chemical across all sources, uses, pathways, and exposure settings to determine whether it poses an unreasonable risk. Using the best available science, as required by TSCA, means EPA must quantify the aggregate exposures and cumulative risks.^53,54^

EPA should combine quantitative exposure estimates across exposure pathways and settings (Figure 1), including chemical uses not subject to TSCA such as food packaging, and assess the impacts of exposure to multiple chemical mixtures and structural drivers of health.^53,25,54,62^ This approach is in line with existing EPA guidance, approaches recommended by authoritative bodies such as the National Academies of Sciences, Engineering, and Medicine (NASEM), and an executive order from President Biden.^53,54,49,63,64^

In January 2022, EPA released a draft screening level methodology to assess air and water exposure pathways for the general population living near facilities reporting through the Toxic Release Inventory.^44^ Although EPA specifically states the case studies are not meant as aggregate or cumulative exposure or risk frameworks, the techniques could be adapted for such. In April 2022, EPA issued its Equity Action Plan, highlighting a commitment to addressing cumulative impacts across its programs.^48^

EPA should acquire the data necessary to conduct aggregate and cumulative assessments by using TSCA’s data gathering and testing authorities (see Data Gaps below). To facilitate timely risk evaluations, EPA should utilize health-protective adjustment factors while more specific data are under development.^28−30^

## Potentially Exposed or Susceptible Subpopulations (PESS)

Exposures to toxic chemicals disproportionately impact the health of groups such as children, low-wealth communities, and communities of color.^16,65−67^ Failure to identify all PESS and account for quantitative differences in risk of susceptible subpopulations results in underestimation of risk.

## What Is Required under Amended TSCA

Under amended TSCA, EPA is mandated to (SI Section 1):

determine whether a chemical substance presents an unreasonable risk of injury to health or the environment, without consideration of costs or other nonrisk factors, including an unreasonable risk to a potentially exposed or susceptible subpopulation. . .§2605(b)(4)(A).

PESS is defined as:

a group of individuals within the general population identified by the Administrator who, due to either greater susceptibility or greater exposure, may be at greater risk than the general population of adverse health effects from exposure to a chemical substance or mixture, such as infants, children, pregnant women, workers, or the elderly §2602(12).

## How EPA Implemented These Requirements

In its proposed risk evaluation framework rule, EPA’s definition of PESS elaborated on the statutory definition to better capture intrinsic and extrinsic factors affecting susceptibility:

Potentially exposed or susceptible subpopulation means a group of individuals within the general population identified by the Agency who, due to either greater susceptibility or greater exposure, may be at greater risk than the general population of adverse health effects from exposure to a chemical substance or mixture, including but not limited to, infants, children, pregnant women, workers, or the elderly. EPA may identify a susceptible subpopulation in an individual risk evaluation upon consideration of various intrinsic (e.g., life stage, reproductive status, age, gender, genetic traits) or acquired (e.g., pre-existing disease, geography, workplace) characteristics that may affect exposure or modify the risk of illness or disease.^68^

In the final risk evaluation framework rule, EPA did not use the language from the proposed rule and instead used the text of the statute (see above and SI Section 1) without elaboration.^33^ This definition does not explicitly identify the full range of intrinsic and extrinsic factors influencing the health impacts of chemical exposures.

EPA’s approach and terminology to identify PESS varied considerably in the first 10 risk evaluations. Among the inconsistencies are differences in whether health conditions related to a chemical’s hazards were considered and whether fenceline communities were included, as detailed below and by other experts.^28^ Additionally, EPA’s language regarding PESS is vague, in some cases discussing general factors that may increase susceptibility (e.g., alcohol consumption, nutrition, genetic differences) without clearly identifying groups as PESS. In several instances, groups named as PESS in the statute were not identified in the risk evaluations; for example, pregnant and aging populations were not considered PESS for 1,4-dioxane and PV-29. SI Section 2, Table S3 illustrates the range of approaches and deficiencies in identifying PESS for four of the first 10 chemical risk evaluations. While EPA’s approaches to identifying PESS varied, its approaches to quantifying PESS risks were consistent. For PESS identified based on elevated exposure, EPA’s used “high-end” estimates of exposure for each condition of use and exposure pathway in calculating risks. EPA said these high-end estimates, which do not consider aggregate exposures, satisfied its statutory requirement regarding sentinel exposures (see Aggregate Exposure and Cumulative Risk section above). For PESS identified as having elevated susceptibility, EPA did not adjust its risk calculations, saying it lacked “sufficient quantitative information about these potential sources of susceptibility.”^69^ EPA used a 10-fold adjustment factor to account for human variability, noting uncertainty regarding whether it was sufficient to account for differences in risk of susceptible subpopulations. However, lack of data does not equate to lack of hazard or risk.^27^

## Implications for Public Health

Scientific evidence demonstrates intrinsic (e.g., age, pre-existing disease, reproductive status, genetics) and extrinsic factors (e.g., stress, racism, poverty, and geographic/socioeconomic/cultural/workplace factors) can increase exposures and susceptibility to environmental chemical exposure risks as well as adverse health outcomes.^70−74^ Communities of color disproportionately bear the burden of adverse health impacts from chemical exposures. Compared to white non-Hispanic children, Black children are more likely to be diagnosed with asthma (14% v. 6.5%) and learning disabilities (10.2% v. 7.9%); and Black women are more likely to experience preterm birth compared to white non-Hispanic women (14% v. 9.2%).^75^ Compared to white non-Hispanic children, Latino children are more likely to be diagnosed with obesity (24% v. 14%) and Puerto Rican children are more likely to be diagnosed with autism (4.6% v. 2.9%).^75^ Contrary to the direction of amended TSCA, EPA did not take a comprehensive and consistent approach to identifying or considering PESS in the first 10 risk evaluations and omitted PESS identified in the statute; ultimately leading EPA to underestimate risk. For identified PESS, EPA did not apply approaches ensuring elevated exposures and risks of these populations were completely accounted for.

The 1-bromopropane risk evaluation is an example of EPA’s limited approach to quantifying risks to PESS. EPA identified a single exposure to this dry cleaning chemical during a critical window of fetal development may be sufficient to produce adverse developmental effects.^37^ However, it “did not calculate risk for children associated with acute exposure at dry cleaners because the acute health domains (developmental effects) are not applicable to children”.^37^ Further, EPA did not calculate risks for chronic exposure for children at dry cleaners because “EPA believes exposure to children at workplaces are unlikely to be chronic in nature”.^37^ EPA’s risk evaluation assumes exposures to children happen only in a 4 h period after school, likely inaccurate for school-age children and younger who may spend the majority of their time in family owned dry-cleaning facilities.^76^

EPA generally accounted for differential dose–response in identified PESS throughout the risk evaluations by assuming the typical 10-fold factor to account for human variability was sufficient to account for any differences. EPA applied this default without evaluating its sufficiency, and despite contrary evidence, overall underestimating risk to PESS.^29,53^

## Recommendations for Change

EPA should explicitly name parameters qualifying populations as susceptible to ensure its risk evaluations assess whether each chemical poses an unreasonable risk to PESS. EPA should use a modified version of the PESS definition from its 2017 proposed TSCA risk evaluation framework rule, explicitly identifying intrinsic and extrinsic factors:

Potentially susceptible subpopulation means a group of individuals or communities within the general population identified by the Agency who, due to greater susceptibility may be at greater risk than the general population of adverse health effects from exposure to a chemical substance or mixture, including but not limited to infants, children, pregnant women, workers, or aging populations. Susceptibility can be due to both intrinsic (e.g., pre-existing disease, life stage, reproductive status, age, sex, genetic traits) and extrinsic (e.g., food insecurity, geography, socioeconomic status, racism/discrimination, cultural, workplace) factors when identifying this population.

EPA should prepare a comprehensive methodology to identify PESS and quantify their risks consistently within and across the TSCA risk evaluations; it has taken this approach in identifying at-risk populations under the Clean Air Act and the NASEM identified this as a goal.^77,78^ Studies by community groups such as the impacts of the Deepwater Horizon oil disaster on fishing communities, the quantification of heavy metals in water used by Native American tribes the consumption of fish by tribal populations in heavily polluted areas, and air pollution in Detroit can be used as guides.^79−82^

EPA should use its data gathering authorities (Data Gaps section below) and quantify the exposure and risks to all PESS, and as data are being developed, EPA should utilize health-protective defaults to account for elevated exposures and susceptibility where specific data are lacking, as recommended by the NASEM.^53^ Data and methods are available for improved treatment of human variability, including probabilistic methods, in cases where chemical-specific data are unavailable.^29,30,53,83,84^

## Data Gaps

The data underpinning risk evaluations must be extensive, multidisciplinary, and sufficient to quantify all relevant hazard end points; failure to do so will understate exposure levels and underestimate risk, particularly for PESS.

## What Is Required under Amended TSCA

Amended TSCA states EPA has broad authority to collect relevant information for the identification, prioritization, risk evaluation, and risk management processes (SI Section 1). It is required that the Administrator:

take into consideration information relating to a chemical substance or mixture, including hazard and exposure information, under the conditions of use, that is reasonably available to the Administrator (§ 2625(k)).

Reasonably available information is defined as:

information that EPA possesses or can reasonably generate, obtain and synthesize for use, considering the deadlines specified in 15 U.S.C. 2605(b) for prioritization and risk evaluation.^33^

EPA has various tools to obtain the data for a comprehensive risk evaluation, including TSCA section 8 which authorizes EPA to require manufacturers and processors to submit reports to EPA containing the volume of the chemical manufactured or processed, the conditions of use and the hazard and exposure potential (§ 2607(a)); submit any records of significant adverse reactions to health or the environment alleged to have been caused by the substance or mixture (§ 2607(c)); and submit unpublished health and safety studies (§ 2607(d)).

When EPA lacks necessary information to perform a risk evaluation, the Administrator may use TSCA section 4 to:

require the development of new information relating to a chemical substance or mixture if the Administrator determines that the information is necessary to. . .perform a risk evaluation under section 2605(b) of this title (§ 2603(a)(2)(A)(i)).

Under amended TSCA, EPA can use its section 8 reporting authorities (§2607) and section 4 testing authority (§2603) to require chemical manufacturers to provide the data, including conducting new health effects studies of chemicals, necessary to perform a risk evaluation.

## How EPA Implemented These Requirements

EPA did not issue any section 4 test orders for toxicity information for the first 10 chemical risk evaluations, despite several chemicals, such as C. I. Pigment Violet 29 (PV29), lacking necessary information on critical health end points.^85^ In conducting the first 10 risk evaluations EPA only used its section 4 authority to issue test orders for PV29, and those test orders were limited to solubility testing and occupational exposure monitoring, without requiring any health effects studies.^85^

## Implications for Public Health

EPA must have sufficient data on health effects and exposures to conduct a comprehensive risk evaluation. However, in several instances, EPA determined conditions of use of the first 10 chemicals evaluated under amended TSCA did not present an unreasonable risk without sufficient information. Peer reviewers in EPA’s Science Advisory Committee on Chemicals (SACC) identified multiple instances of inadequate information, such as “large data gaps that preclude coming to confident conclusions regarding certain subpopulations” (PV29) and “information used to evaluate worker exposure was generally lacking in its ability to present a coherent picture of this critical element of risk” (1,4-dioxane).^86^

EPA did not address critical data gaps even after they were identified in peer review. For example, the SACC found “insufficient data to assess the potential neurotoxicity of 1,4-dioxane. . .[or] to assess the toxicity of 1,4-dioxane on other non-cancer outcomes such as immunotoxicity.”^37^ EPA did not use its statutory authorities to obtain data to assess these outcomes, preventing them from being considered in the final risk evaluation.

Where there was scant data, EPA failed to account for limitations and inappropriately drew conclusions about health effects. For example, EPA determined PV29 was not a reproductive or developmental hazard based on a study conducted using guideline OECD 421. However, the OECD 421 test protocol and EPA’s risk assessment guidelines clearly establish OECD 421 alone cannot show a chemical is not a reproductive or developmental toxicant, and additional data are needed to establish a chemical lacks reproductive/developmental toxicity. Instead, EPA disregarded the test protocol’s established limitations and concluded PV29 did not cause reproductive toxicity, as “EPA believes that OECD 421 is adequate to determine whether additional reproductive testing is necessary. As no significant adverse effects were observed in the study, EPA believes that this provides justification that no additional reproductive testing is necessary.”^87^ Without further testing, however, EPA’s conclusion PV29’s reproductive or developmental toxicity is invalid and does not represent the best available science.

## Recommendations for Change

EPA must apply its reporting and testing authorities under amended TSCA to require chemical manufacturers to provide the data, including toxicity studies, necessary to perform its ongoing and future risk evaluations (SI Section 1). EPA must also implement approaches to incentivize and require manufacturers to provide appropriate and independent data. It is critical EPA increase transparency by reevaluating the confidential business information (CBI) claims allowing industry to shield critical data from public view as more than 50 000 chemicals worldwide have been registered for use without disclosing their identities.^88^ Second, EPA should derive provisional toxicity values, applying multiple default adjustment factors as needed to account for any lack of data, as recommended by authoritative bodies such as the NASEM.^53,89−93^

Third, the application of “New Approach Methods” (NAMs) has been proposed to facilitate the number of hazard evaluations EPA can complete, while replacing the need for animal testing and reducing costs.^94,95^ While there is potential for these tools to provide more timely information on hazards of concern, thus reducing the time between potential human exposure and action to mitigate these harms, NAMs also have well established limitations, including limits to their ability to identify chronic and systemic health end points such as immunotoxicity, endocrine effects and developmental neurotoxicity.^96−98^

These limitations have led the U.S. EPA Children’s Health Protection Advisory Committee (CHPAC) to warn in a recent report that “cell-based assays and other high-throughput toxicity tests, often called New Approach Methods (NAMs), have the potential to provide needed data and could be used to establish potential hazards or upgrade overall hazard identification. However, due to important limitations, data from NAMs cannot be used to rule-out a specific hazard”.^99^ EPA should instead use NAMs to provide “actionable evidence”, or a scientific basis for health protective actions, as recommended by regulatory agencies such as California EPA.^100^

Amended TSCA requires EPA to complete priority designations no more than 12 months after formally initiating the prioritization process for a chemical, and risk evaluations must be completed in 3–3.5 years after a chemical is designated “High Priority” ( SI Section 1, Section 2, and Figure S2). As many studies take multiple years to conduct, the current process does not afford EPA enough time to fill critical data gaps and incorporate new information into the risk evaluation. Thus, EPA must identify these gaps *before* prioritization begins. EPA can implement a “pre-prioritization” process to identify and address data needs necessary for comprehensive risk evaluation, as outlined in the January 2017 proposed prioritization framework rule (SI Section 3).

To implement a preprioritization process, the Agency should regularly update a formal list of candidates for risk evaluation and immediately require TSCA section 8 reporting of existing health and safety information when a chemical is added to the list. After evaluating the section 8 submissions and other reasonably available data, EPA should identify data gaps and issue TSCA section 4 test orders to obtain critical missing information for a comprehensive risk evaluation. This proactive process would ensure EPA can identify and fill data gaps before the 3.5-year process of risk evaluation is initiated.

To accurately assess the health risks posed by chemicals, EPA must ensure the data it requires are comprehensive. To speed data generation, EPA should explicitly define a generic target data set, including physical characteristics, health end points, and PESS considerations, with input from scientific and community experts.^27^ The data set could identify a range of health effects (e.g., cardiovascular, reproductive and neurodevelopmental toxicity, carcinogenesis) across sensitive life stages (e.g., preconception, fetal and child development, aging), with robust and sensitive assays to identify risk of human health effects. This framework for identifying critical data gaps would guide how EPA can use its statutory authorities for each chemical (based on database completeness).^27^ EPA’s task is complicated as amended TSCA places the burden on EPA to identify data gaps and obtain data needed to evaluate chemical risks. Thus, a more health-protective version of TSCA would require chemical manufacturers to provide independent and robust health and environmental assessment data to EPA for their chemicals to remain on the market.

## Systematic Review

Systematic review is an approach to ensure all relevant studies are identified and transparently evaluated using prespecified methods to reduce bias; failure to use appropriate methods can result in exclusion of relevant information concerning exposures and hazards and underestimate risk. Well-established systematic review methods in the field of medicine have been adapted to environmental health.^101−113^

## What Is Required under Amended TSCA

Amended TSCA requires EPA consider the “weight of the scientific evidence,”^19^ (SI Section 1) when making decisions about chemical risks, which EPA defines in the risk evaluation framework rule as:

a systematic review method, applied in a manner suited to the nature of the evidence or decision, that uses a pre-established protocol to comprehensively, objectively, transparently, and consistently identify and evaluate each stream of evidence, including strengths, limitations, and relevance of each study and to integrate evidence as necessary and appropriate based upon strengths, limitations, and relevance.^33^

## How EPA Implemented These Requirements

In 2018, EPA published the *Application of Systematic Review in TSCA Risk Evaluations* (TSCA Method) to “guide the Agency’s selection and review of the scientific studies that are used to inform TSCA chemical risk evaluations”.^114^ The TSCA Method used in the first 10 risk evaluations, diverged from established best practices for systematic review in every of step of the systematic review process (SI Section 1; Section 2, and Figure S3).

A systematic review method establishes *what* evidence EPA considers and *how* it is evaluated when conducting risk evaluations. Publishing a protocol outlining how the assessment will be conducted in advance is an essential initial step. It ensures judgements regarding the approach to study selection (literature search and screening), study evaluation (internal validity and quality of the body of evidence), evidence synthesis (each evidence stream separately) and evidence integration (across human, animal, in vitro streams) are made before reviewing the evidence so knowledge of the results does not bias the risk evaluation. Publication of a *prespecified* protocol is established as a best practice by all valid systematic review methods. EPA explicitly identified a *prespecified* protocol as an element of TSCA systematic review in its risk evaluation framework rule and in the method documentation. However, EPA did not publish *prespecified* protocols for the first 10 risk evaluations, leaving them open to potential bias.

Well-conducted systematic review protocols specify the approach to evaluating risk of bias in studies. Risk of bias is a systematic error or deviation in the true results or inferences of a study due to how a study was designed, conducted, analyzed, or reported that decrease confidence in the results. Risk of bias tools can evaluate exposure and outcome assessment methods in a study. Rather than utilizing an established method for assessing risk of bias, EPA’s TSCA Method introduced a novel method containing three critical issues and was incompatible with the best available science.^101−113^

1. EPA created an arbitrary list of quality metrics and a rating system that excluded studies from further consideration in the risk evaluations when they were rated as “unacceptable for use” due to “serious flaws”.

However, the “serious flaws” EPA’s tool identified were not all related to deficits in the underlying research. One of the 14 quality metrics EPA’s tool marked as a “serious flaw” is statistical power (the likelihood a study will detect an effect) (SI Section 2 and Table S4). Statistical power does not reflect the quality of the research, as a small study can be underpowered but well-conducted and less biased than a larger study.^115^ In addition, small “underpowered” studies can be combined with other studies in a meta-analysis to derive a more reliable estimate of the relationship between an exposure and an outcome.

2. EPA used a quantitative scoring method, assigning arbitrary numerical weights to quality metrics and then summing across metrics to decide whether a study is of “high,” “medium,” or “low” or “unacceptable” quality.

Previous evaluations on the use of “quality scores” found a lack an empirical basis for weighting the metrics and that they were not able to distinguish between studies with a high and low risk of bias.^115,116^ Authoritative guidance on systematic review recommends the use of *qualitative* domain-based ratings without scores combining ratings across domains.^117^

3. EPA’s method included study reporting as one reason for scoring studies “unacceptable for use” across multiple metrics (Metrics 3, 4, 6, 7).^118^ (SI Section 2 and Table S4).

However, it conflates how well a study is reported with how well the research was conducted. The quality of a study’s reporting does not necessarily indicate the quality of the study or the reliability of its results.^119−122^

## Implications for Public Health

EPA’s failure to prespecify its methods via published protocols for the first 10 risk evaluations potentially biased its evaluation of the evidence. For example, EPA published both the literature search and screening strategy and the results of the title and abstract screening of the literature for carbon tetrachloride in June 2017. EPA then conducted full text screening, applying then unknown criteria to exclude references it deemed irrelevant. EPA’s criteria defining the characteristics of relevant studies were not published until May 2018, almost a year after publication of the searches and initial screening.^123^ The timing means development of the criteria and the determination of which studies were included and excluded could have been biased by knowledge of the results of studies found in the literature search.

In addition, the EPA’s method to assess study quality led to exclusion of relevant evidence from risk evaluations. In the risk evaluation for perchloroethylene, EPA excluded 10 studies because of “unacceptable ratings”, five based on reporting and three due to statistical power.^124^ EPA, therefore, excluded evidence based on considerations unrelated to real flaws in the underlying research. Failure to include all the relevant evidence could result in underestimation of risk or misidentification of PESS.

Recently, the NASEM found EPA’s TSCA Method “does not meet the criteria of “comprehensive, workable, objective, and transparent” systematic review method” and found it “to be lacking objectivity at each step, from not using a defined approach to documenting how the problem formulation and protocol are developed. Further examples include inclusion and exclusion criteria that are too broad to identify the evidence, inherent subjectivity within the metrics that make up the evaluation score for study quality”.^117^ The NASEM also found the TSCA Method resulted in “reduced confidence in the findings” of EPA’s risk evaluations.^117^

## Recommendations for Change

EPA should follow the NASEM recommendations and implement a systematic review method compatible with empirically based existing methods and aligns with authoritative definitions of a systematic review, including the Institute of Medicine.^125^ EPA should use a prespecified protocol outlining scientific methods for every step of each systematic review it conducts, should assess risk of bias in the individual studies without numeric scoring, and should not exclude studies based on study quality or reporting quality.

Following the release of the NASEM report in February 2021, EPA announced it would no longer use the TSCA method.^126,127^ A draft document representing EPA’s revised approach to TSCA systematic review was released in December 2021, but the draft failed to address many NASEM recommendations.^128^ In particular, as the draft represents EPA’s approach to the 23 TSCA risk evaluations currently in progress, EPA still does not satisfy the NASEM recommendation for prespecified methods to be peer-reviewed and publicly available before a risk evaluation is started and it continues to use a quantitative study quality approach including an arbitrary list of quality metrics and a rating system that excludes studies from further consideration in the risk evaluations. Thus, the current risk evaluations are potentially biased.^129^

Our review of the first 10 chemical risk evaluations conducted under amended TSCA finds EPA systematically underestimated risks to human health, particularly to PESS. EPA has completed 10 risk evaluations and, despite flawed approaches, still determined there were unreasonable risks for at least 50%, and upward of 97%, of the identified conditions of use across all of them. While it is scientifically appropriate for EPA to revisit several aspects of the first 10 evaluations, the advantages of making corrections or improvements to the risk evaluations must be balanced against the disadvantages of further delays in issuing risk management rules to address unreasonable risk, which would result in continued harmful exposures. Revisions to the first 10 risk evaluations should prioritize improvements that affect the unreasonable risk determinations or provide a stronger foundation for risk management actions. Failure to remedy shortcomings in the first 10 chemical risk evaluations will result in continued systematic underestimation of risk for chemicals currently and still to be evaluated under amended TSCA.

The goals of amended TSCA and EPA policies often aspire to protect health, but their implementation often fails to ensure equitable, socially just safeguards.^130,131^ Using the recommendations in this paper, EPA could implement amended TSCA to use the best available science and advance its commitment to health equity, address harmful industrial chemicals, and *“take into account the distributional consequences of regulations. . .to ensure that regulatory initiatives appropriately benefit and do not inappropriately burden disadvantaged, vulnerable, or marginalized communities”*.^132^

## Supporting Information Available

Referenced sections of statutory language of amended TSCA, Supplemental Figures and Tables referenced in text, and selected changes between EPA’s proposed and final risk evaluation framework rule. The Supporting Information is available free of charge at https://pubs.acs.org/doi/10.1021/acs.est.2c02079.
