# Supplementary material for: Toxic Substances Control Act (TSCA) Implementation: How the Amended Law Has Failed to Protect Vulnerable Populations from Toxic Chemicals in the United States
Source: Environ Sci Technol. 2022 Aug 18;56(17):11969–82. doi: 10.1021/acs.est.2c02079 (PMC9454241; doi:10.1021/acs.est.2c02079)
Supplement: Supplementary file 1 — es2c02079_si_001.pdf [file es2c02079_si_001.pdf]

Toxic Substances Control Act (TSCA) Implementation: How the Amended Law has Failed to Protect Vulnerable Populations from Toxic Chemicals in the United States

Authors:

Swati D.G. Rayasam[1]

Patricia D. Koman[2]

Daniel A. Axelrad[3]

Tracey J. Woodruff[1,4]

Nicholas Chartres[1*]

[1]Program on Reproductive Health and the Environment, Department of Obstetrics, Gynecology and Reproductive Sciences, University of California San Francisco School of Medicine, San Francisco, CA 94143 USA

[2]Environmental Health Sciences, University of Michigan School of Public Health, Ann Arbor, MI 48109 USA

[3]Independent consultant, Washington, DC 20001 USA

[4]Environmental Research and Translation for Health, Department of Obstetrics, Gynecology and Reproductive Sciences, University of California San Francisco School of Medicine, San Francisco, CA 94143 USA

Supporting Information

SECTION 1: REFERENCED STATUTORY LANGUAGE OF AMENDED TSCA BY SECTION **S3**

INTRODUCTION S3
CONDITIONS OF USE AND EXPOSURE PATHWAYS S3
AGGREGATE AND CUMULATIVE EXPOSURES S4
POTENTIALLY EXPOSED OR SUSCEPTIBLE SUBPOPULATIONS S4
DATA GAPS S5
SYSTEMATIC REVIEW S7

SECTION 2: SUPPLEMENTAL FIGURES AND TABLES **S8**

FIGURE S1. OVERVIEW OF EPA'S PROCESS FOR CONSIDERING BOTH NEW AND EXISTING CHEMICALS UNDER AMENDED TSCA. S8
FIGURE S2. THE PROCESS FOR EVALUATING EXISTING CHEMICALS OR CLASSES OF CHEMICALS UNDER AMENDED TSCA. S8
FIGURE S3. HOW EPA'S TSCA METHOD DID NOT MEET THE WELL-ESTABLISHED SCIENTIFIC CRITERIA FOR BEST PRACTICE OF SYSTEMATIC REVIEW FOR EVERY OF STEP OF THE SYSTEMATIC REVIEW PROCESS. S9
TABLE S1. CONDITIONS OF USE EXCLUDED BY EPA IN FIRST 10 TSCA RISK EVALUATIONS S10
TABLE S2. KNOWN EXPOSURE PATHWAYS EXCLUDED FROM FIRST 10 TSCA RISK EVALUATIONS BECAUSE EPA CLAIMS THE PATHWAYS ARE ADDRESSED BY OTHER ENVIRONMENTAL STATUTES S11
TABLE S3. HOW EPA IDENTIFIED POTENTIALLY EXPOSED AND SUSCEPTIBLE SUBPOPULATIONS (PESS) IN FOUR EXAMPLE TSCA RISK EVALUATIONS S12
TABLE S4. EPA'S LIST OF 14 METRICS (OUT OF 20 TOTAL) WITH SCORING OPTIONS THAT MAKE EPIDEMIOLOGICAL STUDIES IN THE FIRST 10 TSCA RISK EVALUATIONS "UNACCEPTABLE FOR USE IN THE HAZARD ASSESSMENT" S14

SECTION 3: SELECTED CHANGES BETWEEN PROPOSED AND FINAL RISK EVALUATION FRAMEWORK RULE **S15**

Section 1: Referenced statutory language of amended TSCA by section

Introduction

§2617. Preemption

(b)(1) Except as provided in subsections (c), (d), (e), (f), and (g), beginning on the date on which the Administrator defines the scope of a risk evaluation for a chemical substance under section 2605(b)(4)(D) of this title and ending on the date on which the deadline established pursuant to section 2605(b)(4)(G) of this title for completion of the risk evaluation expires, or on the date on which the Administrator publishes the risk evaluation under section 2605(b)(4)(C) of this title, whichever is earlier, no State or political subdivision of a State may establish a statute, criminal penalty, or administrative action prohibiting or otherwise restricting the manufacture, processing, distribution in commerce, or use of such chemical substance that is a high-priority substance designated under section 2605(b)(1)(B)(i) of this title.

Conditions of use and exposure pathways

§2602. Definitions

(4) The term "conditions of use" means the circumstances, as determined by the Administrator, under which a chemical substance is intended, known, or reasonably foreseen to be manufactured, processed, distributed in commerce, used, or disposed of.

§2605. Prioritization, risk evaluation, and regulation of chemical substances and mixtures

(b)(4)(F)

(i) integrate and assess available information on hazards and exposures for the conditions of use of the chemical substance, including information that is relevant to specific risks of injury to health or the environment and information on potentially exposed or susceptible subpopulations identified as relevant by the Administrator;

(iv) take into account, where relevant, the likely duration, intensity, frequency, and number of exposures under the conditions of use of the chemical substance;

Aggregate and Cumulative Exposures

§2605. Prioritization, risk evaluation, and regulation of chemical substances and mixtures

(b)(4)(F) In conducting a risk evaluation under this subsection, the Administrator shall—

(ii) describe whether aggregate or sentinel exposures to a chemical substance under the conditions of use were considered, and the basis for that consideration;

(d)(3)(A)(i) The Administrator may declare a proposed rule under subsection (a) to be effective, and compliance with the proposed requirements to be mandatory, upon publication in the Federal Register of the proposed rule and until the compliance dates applicable to such requirements in a final rule promulgated under section 2605(a) of this title or until the Administrator revokes such proposed rule, in accordance with subparagraph (B), if—(i) the Administrator determines that—

(I) - the manufacture, processing, distribution in commerce, use, or disposal of the chemical substance or mixture subject to such proposed rule or any combination of such activities is likely to result in an unreasonable risk of serious or widespread injury to health or the environment before such effective date without consideration of costs or other non-risk factors;

Potentially Exposed or Susceptible Subpopulations

§2602. Definitions

(12) The term "potentially exposed or susceptible subpopulation" means a group of individuals within the general population identified by the Administrator who, due to either greater susceptibility or greater exposure, may be at greater risk than the general population of adverse health effects from exposure to a chemical substance or mixture, such as infants, children, pregnant women, workers, or the elderly.

§2605. Definitions

(b)(4)(A) The Administrator shall conduct risk evaluations pursuant to this paragraph to determine whether a chemical substance presents an unreasonable risk of injury to health or the environment, without consideration of costs or other nonrisk factors, including an unreasonable risk to a potentially exposed or susceptible subpopulation identified as relevant to the risk evaluation by the Administrator, under the conditions of use

Data Gaps

§ 2625(k) Reasonably available information

In carrying out sections 2603, 2604, and 2605 of this title, the Administrator shall take into consideration information relating to a chemical substance or mixture, including hazard and exposure information, under the conditions of use, that is reasonably available to the Administrator.

40 CFR § 702.3 - Definitions

Reasonably available information means information that EPA possesses or can reasonably generate, obtain and synthesize for use, considering the deadlines specified in 15 U.S.C. 2605(b) for prioritization and risk evaluation. Information that meets such terms is reasonably available information whether or not the information is confidential business information that is protected from public disclosure under 15 U.S.C. 2613

§2603. Testing of chemical substances and mixtures

(2)Additional testing authority.—In addition to the authority provided under paragraph (1), the Administrator may, by rule, order, or consent agreement—

(A)require the development of new information relating to a chemical substance or mixture if the Administrator determines that the information is necessary—

(i)to review a notice under section 2604 of this title or to perform a risk evaluation under section

2605(b) of this title;

(ii)to implement a requirement imposed in a rule, order, or consent agreement under

subsection (e) or (f) of section 2604 of this title or in a rule promulgated under section 2605(a) of this title;

§2605. Prioritization, risk evaluation, and regulation of chemical substances and mixtures

(b)

(1)(C) The rulemaking required in subparagraph (A) shall ensure that the time required to make a priority designation of a chemical substance be no shorter than nine months and no longer than 1 year

(2)

(A) Not later than 180 days after June 22, 2016, the Administrator shall ensure that risk evaluations are being conducted on 10 chemical substances drawn from the 2014 update of the TSCA Work Plan for Chemical Assessments and shall publish the list of such chemical substances during the 180 day period.

(B) Not later than three and one half years after June 22, 2016, the Administrator shall ensure that risk evaluations are being conducted on at least 20 high-priority substances and that at least 20 chemical substances have been designated as low-priority substances, subject to the limitation that at least 50 percent of all chemical substances on which risk evaluations are being conducted by the Administrator are drawn from the 2014 update of the TSCA Work Plan for Chemical Assessments.

§2607. Reporting and retention of information

(a)Reports

(1)The Administrator shall promulgate rules under which—

(A)each person (other than a small manufacturer or processor) who manufactures or processes or proposes to manufacture or process a chemical substance (other than a chemical substance described in subparagraph (B)(ii)) shall maintain such records, and shall submit to the Administrator such reports, as the Administrator may reasonably require, and

(B)each person (other than a small manufacturer or processor) who manufactures or processes or proposes to manufacture or process—

(i)a mixture, or

(ii)a chemical substance in small quantities (as defined by the Administrator by rule) solely for purposes of scientific experimentation or analysis or chemical research on, or analysis of, such substance or another substance, including any such research or analysis for the development of a product,

shall maintain records and submit to the Administrator reports but only to the extent the Administrator determines the maintenance of records or submission of reports, or both, is necessary for the effective enforcement of this chapter.

The Administrator may not require in a rule promulgated under this paragraph the maintenance of records or the submission of reports with respect to changes in the proportions of the components of a mixture unless the Administrator finds that the maintenance of such records or the submission of such reports, or both, is necessary for the effective enforcement of this chapter. For purposes of the compilation of the list of chemical substances required under subsection (b), the Administrator shall promulgate rules pursuant to this subsection not later than 180 days after January 1, 1977.

(2) Administrator may require under paragraph (1) maintenance of records and reporting with respect to the following insofar as known to the person making the report or insofar as reasonably ascertainable:

(A)The common or trade name, the chemical identity, and the molecular structure of each chemical substance or mixture for which such a report is required.

(B)The categories or proposed categories of use of each such substance or mixture.

(C)The total amount of each such substance and mixture manufactured or processed, reasonable estimates of the total amount to be manufactured or processed, the amount manufactured or processed for each of its categories of use, and reasonable estimates of the amount to be manufactured or processed for each of its categories of use or proposed categories of use.

(D)A description of the byproducts resulting from the manufacture, processing, use, or disposal of each such substance or mixture.

(E)All existing information concerning the environmental and health effects of such substance or mixture.

(F)The number of individuals exposed, and reasonable estimates of the number who will be exposed, to such substance or mixture in their places of employment and the duration of such exposure.

(G)In the initial report under paragraph (1) on such substance or mixture, the manner or method of its disposal, and in any subsequent report on such substance or mixture, any change in such manner or method.

(c) Records

Any person who manufactures, processes, or distributes in commerce any chemical substance or mixture

shall maintain records of significant adverse reactions to health or the environment, as determined by the Administrator by rule, alleged to have been caused by the substance or mixture. Records of such adverse reactions to the health of employees shall be retained for a period of 30 years from the date such reactions were first reported to or known by the person maintaining such records. Any other record of such adverse reactions shall be retained for a period of five years from the date the information contained in the record was first reported to or known by the person maintaining the record. Records required to be maintained under this subsection shall include records of consumer allegations of personal injury or harm to health, reports of occupational disease or injury, and reports or complaints of injury to the environment submitted to the manufacturer, processor, or distributor in commerce from any source. Upon request of any duly designated representative of the Administrator, each person who is required to maintain records under this subsection shall permit the inspection of such records and shall submit copies of such records.

(d) Health and safety studies

The Administrator shall promulgate rules under which the Administrator shall require any person who manufactures, processes, or distributes in commerce or who proposes to manufacture, process, or distribute in commerce any chemical substance or mixture (or with respect to paragraph (2), any person who has possession of a study) to submit to the Administrator—(1) lists of health and safety studies (A) conducted or initiated by or for such person with respect to such substance or mixture at any time, (B) known to such person, or (C) reasonably ascertainable by such person, except that the Administrator may exclude certain types or categories of studies from the requirements of this subsection if the Administrator finds that submission of lists of such studies are unnecessary to carry out the purposes of this chapter; and(2) copies of any study contained on a list submitted pursuant to paragraph (1) or otherwise known by such person.

Systematic Review

§ 2625 – Administration

(i)Weight of scientific evidence

The Administrator shall make decisions under sections 2603, 2604, and 2605 of this title based on the weight of the scientific evidence.

Section 2: Supplemental Figures and Tables

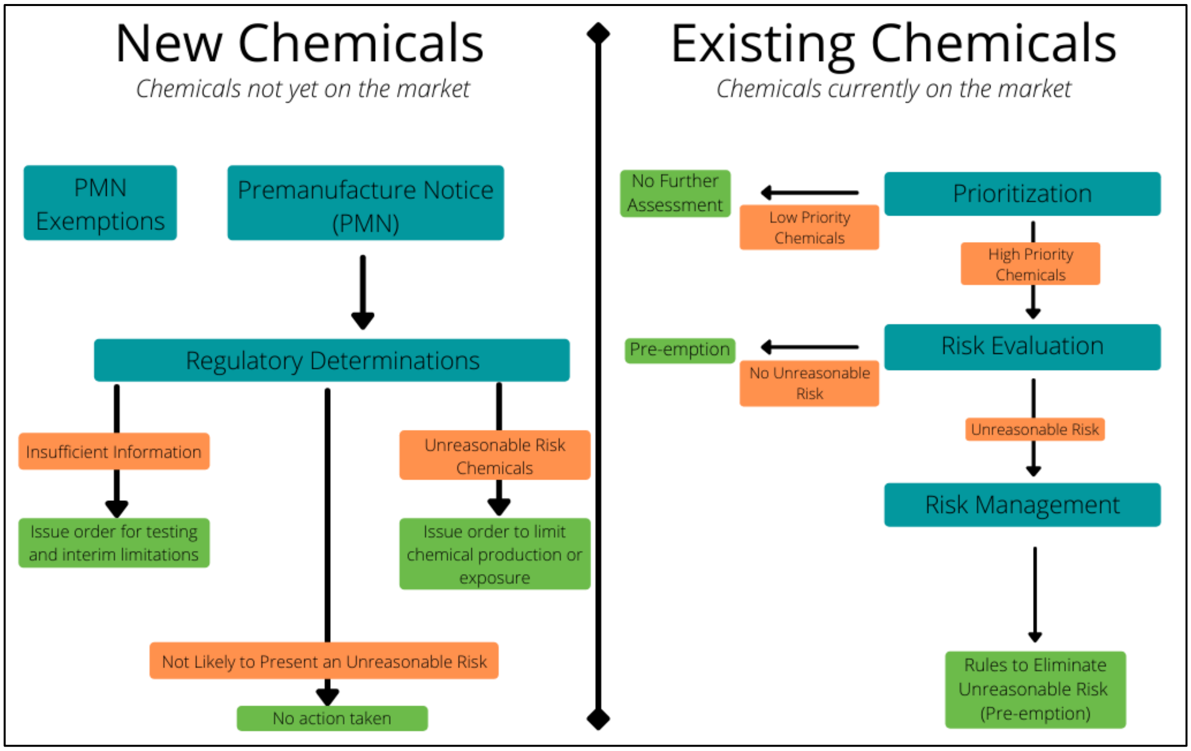

Figure S1. Overview of EPA's process for considering both new and existing chemicals under amended TSCA.

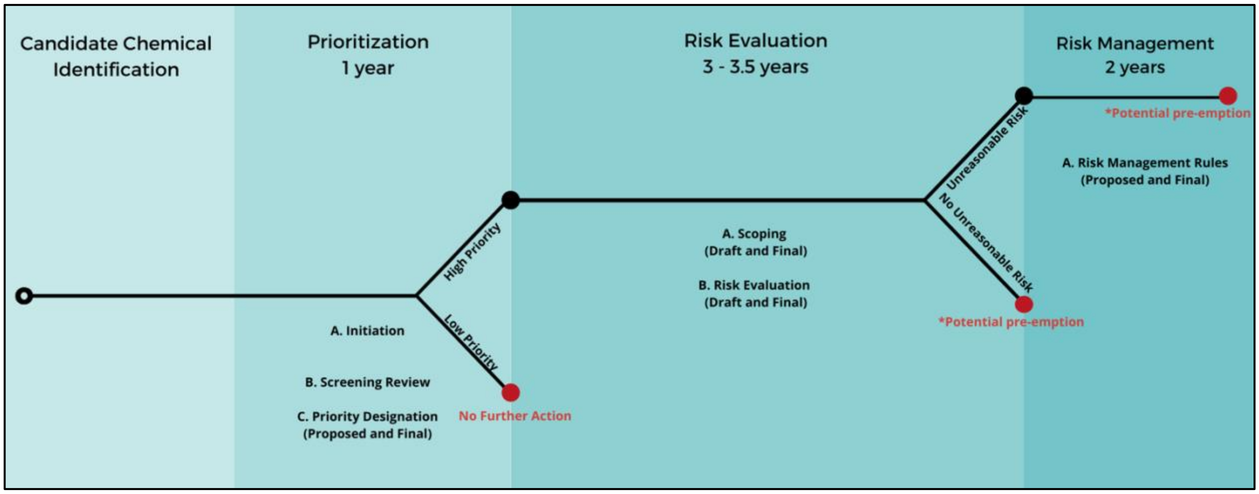

Figure S2. The process for evaluating existing chemicals or classes of chemicals under amended TSCA. After prioritization, EPA has 3.5 years to complete risk evaluations of the high-priority chemicals to determine whether they present an unreasonable risk to health or the environment (proceeding to risk management), or not (no further action will be taken on the chemical). After the initial phase of implementation in which risk evaluation was conducted for the first 10 chemicals, the statute requires that EPA have at least 20 high-priority substance risk evaluations ongoing at all times. *If EPA determines that a chemical presents "no unreasonable risk" then its action may supersede and pre-empt state restrictions. Similarly, EPA's risk management rules for chemicals that pose an "unreasonable risk" can pre-empt state restrictions.

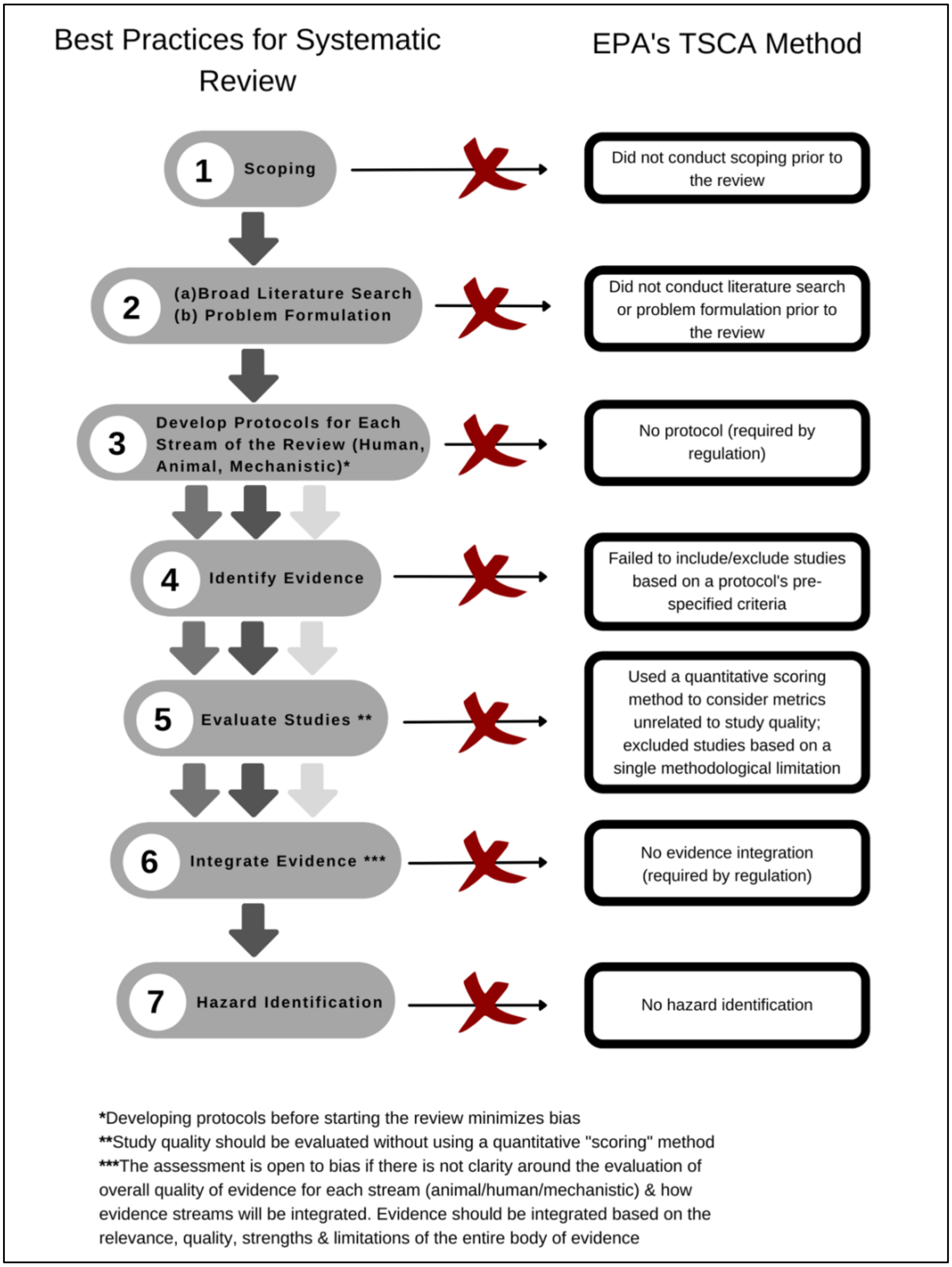

Figure S3. How EPA's TSCA method did not meet the well-established scientific criteria for best practice of systematic review for every of step of the systematic review process. Adapted from Veena I. Singla, Patrice M. Sutton, and Tracey J. Woodruff, 2019: The Environmental Protection Agency Toxic Substances Control Act Systematic Review Method May Curtail Science Used to Inform Policies, With Profound Implications for Public Health American Journal of Public Health 109, 982_984.

Table S1. Conditions of use excluded by EPA in first 10 TSCA risk evaluations[a]

Risk evaluation (chemical)	TSCA conditions of use excluded from scope of risk evaluation
Asbestos	EPA's final "Asbestos Part 1" risk evaluation considers only current uses of asbestos, excluding exposures from legacy uses and associated disposal. Legacy uses are past uses of the chemical, such as asbestos in existing buildings or in existing car brake pads, that would expose workers handling these materials. Associated disposal is future disposals of current legacy uses, such as disposal of asbestos in building products post-demolition or brake pads in a scrapped car. Following a November 2019 court decision that TSCA risk evaluations must include legacy uses,[b] EPA announced it would undertake an "Asbestos Part 2" risk evaluation of legacy uses and associated disposal.[c]
Carbon Tetrachloride	Small amounts of carbon tetrachloride may be present in industrial, commercial and consumer products such as cleaning products and paints. EPA excluded exposures to these products from the risk evaluation, saying it had "a sufficient basis to conclude" that these conditions of use "would present only de minimis exposure or otherwise insignificant risk." No calculations or definitions were provided to support this conclusion.[d]
1,4-Dioxane	1,4-dioxane is an unintended byproduct in the production of ethoxylated chemicals and is in products such as paints, detergents, and antifreeze. Conditions of use related to 1,4-dioxane byproducts were excluded from the draft risk evaluation.[e] In the final risk evaluation this decision was modified, with consumer exposures to 1,4-dioxane byproducts included (with analysis added to the risk evaluation late in the process), but industrial and commercial products and all worker exposures associated with 1,4-dioxane byproducts remained excluded.[f]

[a] Excluded conditions of use described in this table were those explicitly identified by EPA as its exercises of discretion in the final versions of the first 10 risk evaluations. There may be other exclusions based on EPA's asserted discretion identified in other documents supporting the risk evaluations. For example, the response to comments document for TCE states that spills and leaks were excluded from the risk evaluation(s) based on this discretion.[30]

[b]Safer Chems., Healthy Families v. EPA. In *Federal Reporter 3rd Series*, United States Court of Appeals, 9th Circuit: 2019; Vol. 943.

[c]US Environmental Protection Agency. EPA Releases Final Risk Evaluations for Asbestos, Part 1: Chrysotile Asbestos 2020.

[d]US Environmental Protection Agency. Problem Formulation of the Risk Evaluation for Carbon Tetrachloride. 2018. US Environmental Protection Agency. Final Risk Evaluation for Carbon Tetrachloride 2020.

[e] US Environmental Protection Agency. 1,4-Dioxane Draft Risk Evaluation 06-27-2019. 2019.

[f]US Environmental Protection Agency. Final Risk Evaluation for 1,4-Dioxane. 2020.

Table S2. Known exposure pathways excluded from first 10 TSCA risk evaluations because EPA claims the pathways are addressed by other environmental statutes

First 10 Chemical Risk Evaluation	Exposure pathways excluded based on EPA activities under other statutes[a]					
	Ambient air pathway	Drinking water pathway	Ambient water pathway	Land application of biosolids	Onsite releases to land pathway	Disposal pathways
Asbestos	X	X	X		X	X
1-Bromopropane	X[b]				X	X
Carbon tetrachloride	X	X	X	X	X	X
C.I. Pigment Violet 29						
1,4-Dioxane	X	X[c]			X	X
Hexabromocyclododecane						X
Methylene chloride	X	X	X		X	X
n-Methylpyrrolidone	X	X[c]			X	X
Perchloroethylene	X	X	X	X	X	X
Trichloroethylene	X	X	X	X	X	X

[a]Table is drawn from explicit statements in the first 10 risk evaluations regarding pathways that were excluded based on jurisdiction of other statutes administered by EPA.
[b]1-Bromopropane emissions to ambient air are not currently regulated by EPA. In June 2020, EPA announced that it would conduct a rulemaking to add 1-bromopropane to the list of hazardous air pollutants (HAPs) regulated under the CAA. This rulemaking was completed in January 2022, but further rulemakings will be necessary to regulate emissions of 1-bromopropane from any source categories found to emit the chemical.
[c] NMP and 1,4-dioxane in drinking water are not currently regulated by EPA, as EPA has not established Safe Drinking Water Act (SDWA) National Primary Drinking Water Regulations for these chemicals. NMP and 1,4-dioxane are both on the SDWA Candidate Contaminant List, but as of March 2020 EPA concluded that available data were not sufficient to determine whether regulation under SDWA was warranted.

Table S3. How EPA identified potentially exposed and susceptible subpopulations (PESS) in four example TSCA risk evaluations

First 10 Chemical	Groups identified as PESS by EPA	Examples of groups not identified as PESS	Comments
**1-Bromopropane**	• Women of reproductive age and their offspring<br>• Children<br>• Workers<br>• Consumers	• People with pre-existing disease<br>• People >65 yrs old<br>• Fenceline communities[a]	• EPA noted certain lifestages or populations with genetic differences may be PESS due to differential metabolism, but that it did not have sufficient information to identify those groups.[b]<br>• No PESS were identified based on health conditions related to the hazards of the chemical, such as liver toxicity, kidney toxicity, reproductive toxicity, developmental toxicity, and neurotoxicity.
**1,4-dioxane**	• Workers<br>• Consumers<br>• People who recreate in contaminated surface waters<br>• People with liver disease	• Infants<br>• Children<br>• Pregnant women<br>• People with pre-existing disease (other than liver disease)<br>• People >65 yrs old<br>• Fenceline communities	• People with liver disease were identified as PESS, but people with kidney, neurological or respiratory conditions (all identified hazards in the risk evaluation) were not identified as PESS.<br>• EPA's rationale for not identifying pregnant women as PESS is that "There is limited data on reproductive and developmental toxicity."[c]

**Hexabromocyclododecane (HBCD)**	• Pregnant women and women of reproductive age<br>• Infants and young toddlers<br>• Subsistence fishers<br>• People living close to a facility with HBCD releases<br>• Workers using HBCD<br>• People with a high-fat diet and people with elevated body fat<br>• People "with pre-existing health conditions or genetic predispositions related to any of the affected health domains."	• People with specific pre-existing health conditions<br>• People >65 yrs old<br>• Consumers?	• People with pre-existing health conditions were mentioned as PESS, but no health conditions were identified. Thyroid and liver effects were identified as hazards, but people with thyroid or liver conditions were not identified as PESS. The SACC review of the draft risk evaluation stated that there was a "need to…add consideration of several preexisting health conditions that result in higher fat content in the liver." [56, 57]<br>• Not clear from the risk evaluation whether consumers were considered PESS.
**C.I. Pigment Violet 29**	• Workers<br>• Consumers	• Children<br>• Pregnant women<br>• People with pre-existing disease<br>• People aged >65 yrs<br>• Fenceline communities	• EPA did not identify any PESS based on susceptibility to health effects, concluding that "there is no evidence of increased susceptibility for any single group relative to the general population."[57c]

[a] In EPA's draft fenceline screening methodology, EPA identifies 14 out of 15 air exposure scenarios that have increased risk for populations within 10,000 meters of TRI facilities. This document was undergoing peer and public review.

[b] This assumption has no scientific justification. EPA should derive provisional toxicity values, applying multiple default adjustment factors as needed to account for any lack of data, as recommended by authoritative bodies such as the NASEM.

[c] This assumption has no scientific justification, as lack of data does not equate to lack of risk, as highlighted by authoritative bodies such as the NASEM.

Table S4. EPA's list of 14 metrics (out of 20 total) with scoring options that make epidemiological studies in the first 10 TSCA risk evaluations "unacceptable for use in the hazard assessment"*

**Domain**	**Metric**
Domain 1. Study Participation	Metric 1. Participant selection (selection, performance biases)<br>Metric 2. Attrition (missing data/attrition/exclusion, reporting biases)<br>Metric 3. Comparison Group (selection, performance biases)
Domain 2. Exposure Characterization	Metric 4. Measurement of Exposure (Detection/measurement/information, performance biases)<br>Metric 5. Exposure levels (Detection/measurement/information biases)<br>Metric 6. Temporality (Detection/measurement/information biases)
Domain 3. Outcome Assessment	Metric 7. Outcome measurement or characterization (detection/measurement/information, performance, reporting biases)
Domain 4. Potential Confounding/Variable Control	Metric 9. Covariate Adjustment (confounding)<br>Metric 10. Covariate Characterization (measurement/information, confounding biases)
Domain 5. Analysis	Metric 12. Study Design and Methods<br>Metric 13. Statistical power (sensitivity)
Domain 6. Other (if applicable) Considerations for Biomarker Selection and Measurement	Metric 16. Use of Biomarker of Exposure (detection/measurement/information biases)<br>Metric 17. Effect biomarker (detection/measurement/information biases)<br>Metric 20. Sample contamination (detection/measurement/information biases)

*As shown in "Updates to the Data Quality Criteria for Epidemiological Studies" in the Risk Evaluation for Perchloroethylene. Note that metrics 3, 4, 6, and 7 are evaluated using reporting guidelines that are not related to real flaws in the underlying research.

Section 3: Selected changes between proposed and final risk evaluation framework rule

**Risk Evaluation Concept**	**Proposed Rule Text**	**Final Rule Text**
Best available science (definition)	N/A: issue was not addressed in the regulatory text of the proposed framework rule	*Best available science* means science that is reliable and unbiased. Use of best available science involves the use of supporting studies conducted in accordance with sound and objective science practices, including, when available, peer reviewed science and supporting studies and data collected by accepted methods or best available methods (if the reliability of the method and the nature of the decision justifies use of the data).
Conditions of use (definition)	N/A: issue was not addressed in the regulatory text of the proposed framework rule	*Conditions of use* means the circumstances, as determined by the Administrator, under which a chemical substance is intended, known, or reasonably foreseen to be manufactured, processed, distributed in commerce, used, or disposed of. [Definition from the Statute]
Potentially exposed or susceptible subpopulation (definition)	*Potentially exposed or susceptible subpopulation* means a group of individuals within the general population identified by the Agency who, due to either greater susceptibility or greater exposure, may be at greater risk than the general population of adverse health effects from exposure to a chemical substance or mixture, including but not limited to, infants, children, pregnant women, workers, or the elderly. EPA may	*Potentially exposed or susceptible subpopulation* means a group of individuals within the general population identified by the Agency who, due to either greater susceptibility or greater exposure, may be at greater risk than the general population of adverse health effects from exposure to a chemical substance or mixture, such as infants, children, pregnant women, workers, or the elderly. [Definition from the Statute]

	identify a susceptible subpopulation in an individual risk evaluation upon consideration of various intrinsic (*e.g.,* life stage, reproductive status, age, gender, genetic traits) or acquired (*e.g.,* pre-existing disease, geography, workplace) characteristics that may affect exposure or modify the risk of illness or disease.	
Weight of scientific evidence (definition)	N/A: issue was not addressed in the regulatory text of the proposed framework rule	*Weight of scientific evidence* means a systematic review method, applied in a manner suited to the nature of the evidence or decision, that uses a pre-established protocol to comprehensively, objectively, transparently, and consistently, identify and evaluate each stream of evidence, including strengths, limitations, and relevance of each study and to integrate evidence as necessary and appropriate based upon strengths, limitations, and relevance.
Scope of the risk evaluation – conditions of use	*Scope of the risk evaluation.* EPA will determine the scope of the risk evaluation to be conducted for each chemical substance based on all of the following:<br><br>(1) EPA will identify those uses that constitute the conditions of use that will be assessed during the risk evaluation. Those uses shall be all circumstances under which the Agency determines that the chemical substance is intended, known, or reasonably foreseen to be manufactured, processed, distributed in commerce, used, or disposed of.	*Scope of the risk evaluation.* The scope of the risk evaluation will include all the following:<br><br>(1) The condition(s) of use, as determined by the Administrator, that the EPA plans to consider in the risk evaluation.
